# Food noise among U.S. adults: Psychometric evaluation and prevalence estimates from a nationally representative sample

**DOI:** 10.21203/rs.3.rs-10997388/v1

**Published:** 2026-10-09

**Authors:** Branislav Jovanovic, Nickolas Jones, Fengqing Zhang, Alison Holman, Michael Lowe

**Affiliations:** Drexel University; University of California, Irvine; Drexel University; University of California, Irvine; Drexel University

## Abstract

**Background:**

Widespread use of GLP-1 medications has revealed not only their effectiveness in reducing food intake and body mass, but also the clinical relevance of a seemingly underrecognized cognitive-affective process, food noise, that may be highly distressing for some individuals. Although the concept has gained increasing attention in public discourse, empirical research on food noise remains quite limited. We aimed to provide an initial epidemiologic characterization of food noise among U.S. adults.

**Methods:**

Our study utilized data from a nationally representative sample of U.S. adults (*N*=3 646) conducted in Fall 2025. Survey-weighted exploratory and confirmatory factor analyses were used to evaluate the structure of the Food Noise Questionnaire (FNQ; ref. 1). Food noise endorsement was estimated using three operational definitions. Survey-weighted linear regression was used to examine associations between food noise and sociodemographic and weight-related subgroups.

**Results:**

Exploratory factor analysis supported a strongly loaded one-factor solution explaining approximately 80% of the variance. Confirmatory factor analysis supported the one-factor structure, with factor loadings and model fit broadly consistent with the original FNQ validation study. Depending on the operational definition, approximately 4% to 9.5% of U.S. adults met criteria for food noise endorsement. Among adults with obesity, prevalence ranged from approximately 6% to 14%. Food noise scores were higher among women than men (*B*=0.18, 95%CI[0.09, 0.26], *p*<.001), higher among younger adults, and progressively increased across weight categories, from overweight (*B*=0.23, 95%CI[0.13, 0.32], *p*<.001) to class III obesity (*B*=0.61, 95%CI[0.46, 0.76], *p*<.001), relative to normal weight.

**Conclusions:**

These findings provide an initial indication of the potential scope and public health relevance of food noise, suggesting that its occurrence is not negligible but is far from ubiquitous among adults with obesity. Further research is needed to establish clinically meaningful thresholds, determine the overlap of food noise with related constructs, and examine its underlying mechanisms.

## INTRODUCTION

Obesity, whether defined as a body mass index (BMI) ≥ 30 or via clinical criteria (ref. 2), is a complex and heterogeneous condition that affects approximately 40% of US adults (ref. 3) and is associated with substantial public health consequences (refs. 2, 4). A recent breakthrough in obesity treatment involving the use of Glucagon-like peptide 1 (GLP-1) agonists to effectively reduce food intake, weight, and appetite has revealed a potentially novel and underrecognized phenomenon in obesity science: the construct of food noise. Unlike many constructs that emerge through incremental, hypothesis-driven research, food noise was first recognized through consistent patient reports of a dramatic reduction in persistent food-related thoughts following initiation of GLP-1 treatment (ref. 5). These observations suggest that this chronic cognitive-affective process may have been deeply embedded within the lived experiences of individuals with obesity, yet remained obscure and insufficiently examined because it was so normalized and ubiquitous. It was only when GLP-1 medications attenuated this experience, that the phenomenon became clearly evident. In this sense, GLP-1 treatment may not only reduce appetite but also reveal a previously obscured psychological dimension of obesity that may serve as a central mechanism contributing to excess energy intake, dysregulated eating, and reduced effectiveness of interventions for obesity. Consequently, systematically investigating food noise is both timely and necessary.

Food noise has recently been defined as “persistent thoughts about food that are perceived by the individual as unwanted and/or dysphoric and may cause harm, including social, mental, or physical problems” (ref. 5). Common components of food noise include persistent food-related cognitions (i.e., preoccupation with food), difficulty disengaging from these thoughts (i.e., persistence), and associated negative affect (i.e., dysphoria) (refs. 1, 5, 6). However, it remains unclear whether food noise represents a novel construct or a new label for existing constructs in the dysregulated eating literature, such as hedonic hunger (ref. 7), food craving (ref. 8), food preoccupation (ref. 9), and food-cue reactivity (ref. 10). To date, the emerging literature on food noise has been dominated by conceptual, theoretical, and perspective articles (refs. 5, 10-16) focused on defining the phenomenon, examining its conceptual overlap with related constructs, proposing underlying mechanisms, and considering its clinical significance. Empirical research remains limited and has primarily focused on efforts to define and measure food noise despite these conceptual ambiguities. Two early-stage inventories have been developed, including a 23-item and a 7-item Ro Allison Indiana Dhurandhar Food Noise Inventory (RAID-FN), which asses three dimensions: food preoccupation, persistence (i.e., difficulty controlling food-related thoughts), and dysphoria (ref. 6); and a 5-item Food Noise Questionnaire (FNQ) capturing pervasive food preoccupation, perceived lack of control, cognitive burden, and negative emotional and functional impacts, which have been reported to load onto a single latent factor representing food noise (ref. 1). These early measurement efforts suggest that food noise can be operationalized, but its factorial invariance and validity have not yet been established across large or nationally representative samples.

Despite increasing interest in food noise, we still do not know anything about its epidemiology. In other words, it is unclear how food noise is distributed within the U.S. adult population, whether it is commonly experienced, or whether its occurrence differs across demographic groups. The only publicly available estimates come from a consumer-like report published by the WeightWatchers and STOP Obesity Alliance, which estimated that 57% of people with obesity experienced food noise that interfered with adherence to exercise or nutrition plans (ref. 17). However, the report was not peer-reviewed, and its methodology was not reported. Consequently, credible estimates of food noise, let alone population-based estimates, are currently unavailable.

This study aims to provide an initial epidemiologic characterization of food noise among U.S. adults. To accomplish this objective, we first evaluated the psychometric properties of the existing food noise measure (ref. 1) in a nationally representative sample. We then characterized the distribution of food noise by estimating endorsement using multiple thresholds and examining demographic differences across key population subgroups.

## METHODOLOGY

### Study sample and procedures

This cohort study and its statistical analysis plan were pre-registered before data analyses began (https://osf.io/6rp8h/overview). Respondents were drawn from the probability-based NORC AmeriSpeak Panel (ref. 18) as part of an ongoing U.S. national longitudinal study (ref. 19). At baseline (Wave 1; March 2020), 6 514 participants completed the survey and were invited to subsequent surveys. The present cross-sectional study uses data from the Fall 2025 health assessment, which included the food noise measure and other variables relevant to the current analyses.

Surveys were administered online between August 12 and October 6, 2025. Of the 4 087 participants who responded to the Fall 2025 survey, 61 were excluded for data-quality concerns, resulting in a final sample of 4 026 participants. Since the use of GLP-1 medications may influence food-related thoughts and appetite, participants who reported current use of a GLP-1 medication were excluded from the present study. Of the 4 026 participants who completed the Fall 2025 survey, 380 participants reported GLP-1 medication use and were excluded, resulting in a final analytic sample of 3 646 participants.

Survey-weighted analyses adjusted for the probability of selection into the AmeriSpeak Panel among non-GLP users and accounted for differences between this sample and U.S. Census benchmarks for age, sex, race/ethnicity, education, Census region, and attrition over time (ref. 18). NORC reported minimal conditioning effects in the AmeriSpeak panel (refs. 19-20). All study procedures were approved by the Institutional Review Board of the University of California, Irvine.

### Assessments/Measures

#### Food Noise

Food noise was assessed using the 5-item Food Noise Questionnaire (FNQ), which evaluates the level of food noise symptoms over the past two weeks (ref. 1). In addition to being the only validated measure available at the time of survey development, the FNQ demonstrated strong early-stage psychometric properties in the validation study (ref. 1), including excellent internal consistency, high test-retest reliability, a unidimensional factor structure with good model fit, and evidence of convergent and discriminant validity. Items were rated on a Likert-type scale ranging from strongly disagree (1) to strongly agree (5), and assess persistent food-related thoughts, perceived lack of control over these thoughts, time spent thinking about food, interference with daily activities, and negative effects of food-related thoughts on the individual or their life. The full FNQ items are provided in the Supplemental Material. A composite food noise score was calculated as the mean of the five items (range: 1 to 5), with higher scores indicating greater food noise.

### Sociodemographic and Weight Status Characteristics

Sociodemographic categories included age, gender, race/ethnicity, household income, and educational attainment. Body mass index (BMI), calculated as weight in kilograms divided by height in meters squared (kg/m^2^), was used as an imperfect proxy for classifying weight status (ref. 22). Weight status was categorized according to standard clinical thresholds (ref. 23) as underweight (< 18.5 kg/m^2^), normal weight (18.5 to < 25 kg/m^2^; reference group), overweight (25 to < 30 kg/m^2^), obesity class I (30 to < 35 kg/m^2^), obesity class II (35 to < 40 kg/m^2^), and obesity class III (≥ 40 kg/m^2^).

### Statistical Analysis

All analyses were conducted in Stata (ref. 24) and R (v4.4.1; ref. 25), including the *psych*, *lavaan*, and *survey* packages. Prior to conducting the primary analyses, descriptive statistics were computed for all relevant variables.

To replicate the validation of the existing 5-item Food Noise Questionnaire (ref. 1), the nationally representative sample of U.S. adults was randomly divided into two approximately equal subsamples (50/50), with study-specific survey weights retained for all participants. The first half of the sample was used for exploratory factor analysis (EFA), and the second half for confirmatory factor analysis (CFA).

### Factor analyses:

Exploratory factor analysis (EFA) was conducted in the first subsample to evaluate the underlying factor structure and item loadings of the 5-item Food Noise Questionnaire (ref. 1).

Given the ordinal response options of the questionnaire items, factor analyses were based on polychoric correlations (ref. 26). Prior to factor extraction, the inter-item correlation matrix was examined to assess associations among items, and Bartlett's test of sphericity and the measure of sampling adequacy (MSA) were used to assess the suitability of the data for factor analysis (refs. 27-28). The number of factors to retain was evaluated using commonly recommended criteria, including the Kaiser criterion (eigenvalues > 1), visual inspection of the scree plot, and parallel analysis (refs. 27-28). A survey-weighted EFA was additionally examined as a sensitivity analysis.

Following the EFA, a survey-weighted CFA was conducted in the second subsample to evaluate the factor structure identified in the exploratory analysis. Since the questionnaire items were ordinal, CFA models were estimated using the weighted least squares mean- and variance-adjusted (WLSMV) estimator, which is appropriate for ordinal items (refs. 29-30). Model fit was evaluated using the standard metrics (refs. 31-32), such as chi-square statistic, Comparative Fit Index (CFI), Tucker-Lewis Index (TLI), Root Mean Square Error of Approximation (RMSEA), and Standardized Root Mean Square Residual (SRMR). The weighted CFA was subsequently repeated using the full sample. Factor loadings and model fit estimates were also compared descriptively with the original validation study (ref. 1).

### Operational definition for estimating the prevalence of food noise:

Following evaluation of the factor structure, the proportion of participants meeting criteria for endorsement of food noise was estimated using three operational definitions. First, food noise was considered present when the mean score across the five items was ≥ 4, corresponding to an average response of “Agree” or higher. Two additional definitions used item-level criteria, classifying food noise as present when at least 3 of 5 items or at least 4 of 5 items were endorsed at the level of “Agree” or higher. The prevalence of food noise was additionally estimated separately among participants with and without obesity (BMI ≥ 30 kg/m^2^), using each of the three operational definitions and incorporating survey weights.

### Sociodemographic correlates of food noise:

Survey-weighted ordinary least-squares regression analyses were then conducted to examine associations between food noise and demographic characteristics (e.g., age, gender, race/ethnicity, household income, and educational attainment). Additional analyses adjusted for weight status as determined by BMI.

## RESULTS

### Sample characteristic

Characteristics of the 3 646 participants included in the analytic sample are presented in Table S1 (Supplemental Material). The weighted sample was 53.9% female. Approximately, 11% of participants were between 18 and 29 years old, a third and a quarter of the sample were 30–44, and 45–59 years old, respectively, while 30.1% of participants were 60 years or older. Slightly more than 60% of the participants identified as White, non-Hispanic, and household income was relatively evenly distributed across the four income categories. Participants were classified as having underweight (1.7%), normal weight (26.9%), overweight (30.6%), or obesity (40.8%).

### Distributions of different food noise items

Figure 1 show the distribution of responses to the five Food Noise Questionnaire items, while Figures S1-S10 show these distributions across different demographic groups. Across the five FNQ items, endorsement ranged from 6% to 15%, with the highest endorsement observed for constantly thinking about food throughout the day (15%) and the lowest for food-related thoughts being distracting (6%).

### Validation of the FNQ items

As shown in Table 1, the suitability of the FNQ items for factor analysis was examined and confirmed using the inter-item correlations (0.68–0.86), the Kaiser-Meyer-Olkin (KMO) measure of sampling adequacy (MSA = 0.90), and a statistically significant Bartlett’s test of sphericity (*p*<.001). An unweighted EFA based on the polychoric correlation matrix was conducted to examine the factor structure. The number of factors was evaluated using the Kaiser criterion (eigenvalues > 1), visual inspection of the scree plot, and parallel analysis. Although parallel analysis suggested consideration of a two-factor solution, the Kaiser criterion and scree plot supported a one-factor solution. Specifically, the first eigenvalue (4.19) was substantially larger than the remaining eigenvalues (0.13–0.38), with a clear drop-off after the first factor (Figure S11). In addition, the two-factor solution did not yield a substantively interpretable factor structure, with one item demonstrating substantial cross-loadings across both factors. All five items loaded strongly on the single factor, with loadings ranging from 0.80 to 0.93, and accounting for 79.9% of the variance. As a sensitivity analysis, a survey-weighted EFA was also examined, yielding a consistent one-factor structure and similar item loadings.

The one-factor structure identified in the EFA was then evaluated using a survey-weighted CFA in the validation subsample. All five items demonstrated strong standardized factor loadings, ranging from 0.80 to 0.92 (Table 2). Model fit was generally supportive of the proposed one-factor structure, with CFI = 0.98, TLI = 0.95, and SRMR = 0.02. The RMSEA was 0.15; however, RMSEA can be less reliable in models with very few degrees of freedom (ref. 33). The same one-factor model was subsequently estimated in the full sample, yielding similarly strong factor loadings (0.81–0.92) and comparable model fit (CFI = 0.97, TLI = 0.95, SRMR = 0.02, RMSEA = 0.17). The consistency of the factor loadings and other fit indices across the validation and full samples provided support for the proposed one-factor structure of the Food Noise Questionnaire. Table 2 shows the factor loadings and the CFA fit indices from the present study alongside those reported in the original FNQ validation study.

Finally, the FNQ demonstrated high internal consistency, with a Cronbach’s *α* of 0.92.

### Distribution and prevalence of food noise

Figure S12 shows the distribution of the mean food noise scores which ranges from 1 (strongly disagree) to 5 (strongly agree). Food noise prevalence varied according to the threshold used to define endorsement (Table 3). In the least stringent criterion (≥ 3 of 5 items endorsed as “Agree” or “Strongly agree”), 9.46% of the U.S. adults met criteria for food noise. This proportion decreased to 6.12% when endorsement of ≥ 4 of 5 items was required. Using the most stringent criterion, 3.97% of participants met criteria for food noise based on a mean score of ≥ 4 (“Agree”) across all five items. Among adults with obesity, food noise prevalence ranged from 6.05% to 13.67%, depending on the operational criterion (see Table 3). Finally, approximately a quarter of U.S. adults endorsed at least one item, including approximately a third of those with obesity and a fifth of those without obesity (Table S2).

### Sociodemographic correlates of food noise

Table 4 presents the results of two survey-weighted cross-sectional linear regression models examining associations between food noise and demographic characteristics (Model A) and demographic characteristics with additional adjustment for weight status (Model B). In Model A, younger age and lower educational attainment were associated with higher mean food noise scores. Compared with participants older than 60 years, food noise scores were higher among participants that were 18–29 years (*B* = 0.50, 95%CI[0.33, 0.67], *p*<.001), 30–44 years (*B* = 0.35, 95%CI[0.26,0.45], *p*<.001), and 45–59 years old (*B* = 0.17, 95%CI[0.08, 0.26], *p*<.001). Men had lower food noise scores compared to women (*B* = − 0.19, 95%CI[−0.28, −0.11], *p*<.001). Lower educational attainment was also associated with higher food noise, with high school graduates reporting higher scores than those with a university degree (*B* = 0.17, 95%CI[0.04,0.29], *p*=.008).

In Model B, we also adjusted for weight status defined by the BMI. The associations between age and gender and mean food noise scores remained largely unchanged. Compared with participants with normal weight, those classified as overweight (*B* = 0.23, 95%CI[0.13, 0.32], *p*<.001), obesity class I (*B* = 0.35, 95%CI[0.23, 0.47], *p*<.001), obesity class II (*B* = 0.52, 95%CI[0.34, 0.69], *p*<.001), and obesity class III (*B* = 0.61, 95%CI[0.46, 0.76], *p*<.001) had progressively higher mean food noise scores. The association was not statistically significant for participants classified as underweight (*B* = 0.11, 95%CI[−0.29, 0.51], *p*=.587). Finally, Figure S13 presents the results of post hoc logistic regression analyses using three different operationalizations of food noise endorsement; the findings were consistent with those of the survey-weighted cross-sectional linear regression models.

## DISCUSSION

In this initial epidemiologic characterization of food noise among a representative sample of U.S. adults, we evaluated the psychometric properties of the 5-item Food Noise Questionnaire (ref. 1), estimated the prevalence of endorsing food noise using multiple operational definitions, and examined differences in food noise across demographic and weight groups. The FNQ demonstrated a strong and reproducible one-factor structure, with all five items loading strongly on a common latent factor and factor loadings and model fit indices that were consistent with those reported in the original validation study. Depending on the operational definition used, an estimated 4.14% to 9.46% of U.S. adults not currently taking GLP-1 medications met criteria for food noise endorsement. The prevalence was higher within adults with obesity, ranging from approximately 6% to almost 14% depending on the criterion. Food noise scores were higher among younger adults and women and increased progressively across BMI categories. On the other hand, food noise was not consistently associated with race/ethnicity, household income, or educational attainment.

Food noise is an emerging cognitive-affective construct characterized by persistent food-related thoughts, difficulty disengaging from these thoughts, and associated negative affect (ref. 5). Despite growing attention to food noise in both public discourse and recently in the scientific literature, important questions remain about its conceptualization and clinical relevance. Specifically, it is unclear whether food noise represents a distinct construct or reflects an amalgamation of previously studied processes (refs. 7-10). Further, its prevalence, underlying mechanisms, and position within the broader literature on dysregulated eating remain poorly understood. Other conceptual, methodological, and ethical considerations have also been raised (ref. 12). Addressing these questions is important for determining whether food noise represents a theoretically and clinically meaningful construct that warrants further study.

Our findings provide additional support for the psychometric properties of the 5-item FNQ in a large, nationally representative sample and its ability to capture a common underlying dimension of food noise. This unidimensional structure of the FNQ (ref. 1) should be interpreted together with the emerging evidence from the RAID-FN (ref. 6), which has identified three factors in both its longer and shorter forms (i.e., food preoccupation, dysphoria, persistence). The one-factor solution observed for the FNQ may reflect its limited number of items, which could constrain the detection of potentially distinguishable dimensions of food noise. However, the convergence of several features of food noise within a single factor raises the possibility that the FNQ may capture an overarching experience while obscuring psychometrically or clinically meaningful distinctions among its component dimensions. The 5-item FNQ may therefore provide a parsimonious and integrative framework for capturing a constellation of persistent, intrusive food-related thoughts, associated distress, and ruminative persistence within a single, cohesive, and easily administered measure. Such an integrative approach may capture aspects of food noise that are not fully represented by existing, more narrowly defined constructs, with the potential to better explain and predict dysregulated eating outcomes. On the other hand, this consolidated approach may come at the expense of specificity. For example, collapsing potentially distinct dimensions (e.g., food preoccupation, persistence) into a single construct may limit our ability to distinguish clinically meaningful differences among dimensions of food noise, and their relationship with related constructs. Further research is needed to determine whether greater dimensional specificity in food noise is warranted, and, importantly, whether food noise provides incremental validity beyond established related constructs (e.g., craving).

Depending on the operational definition, the prevalence of food noise endorsement ranged from 4.14% to 9.46% among U.S. adults and from 6.05% to 13.67% among U.S. adults with obesity. These estimates suggest that food noise is not a negligible phenomenon, particularly given the psychopathological significance of the items it encompasses, and the relatively stringent criteria used to define endorsement. To clarify, each FNQ item reflects a potentially maladaptive dimension of food noise, including persistent food preoccupation, perceived lack of control, excessive cognitive burden, and negative emotional and functional impacts. Although endorsement of a single item does not denote a clinically meaningful impairment or distress, approximately a third of U.S. adults with obesity and a fifth of those without obesity endorsed at least one FNQ item, suggesting that psychological burden of living in the obesogenic food environment may be substantial, particularly at the population-level. At the same time, our data suggests that food noise is far from ubiquitous among adults with obesity, despite its growing recognition in public discourse. We know of no other studies that have established an empirically and clinically meaningful threshold for food noise. Therefore, our estimates should be interpreted as preliminary estimates of population-level endorsement rather than a prevalence of a clinically established construct. These findings provide an initial indication of the potential population scope and public health relevance of food noise.

Finally, examination of demographic and weight-related subgroups revealed that food noise endorsement was not consistently associated with race/ethnicity or socioeconomic variables (i.e., household income, education), after accounting for BMI. In contrast, food noise scores were higher among women and younger adults. The observed decline in food noise with age raises questions about whether these differences represent individual-level variation or cohort differences in exposure to the obesogenic food environment. These sociodemographic associations are consistent with findings from the original FNQ validation study (ref. 1), but differ from those of the RAID-FN study, which reported no significant differences across these groups, including age and sex (ref. 6).

Our study also demonstrated that food noise monotonically increased across BMI-defined weight categories. This finding extends prior evidence from Diktas and colleagues (ref. 1), who observed a positive linear association between BMI and FNQ scores, but did not detect significant differences in FNQ scores across BMI-defined categories. In contrast, Dhurandhar and colleagues (ref. 6) reported no significant correlation between BMI and food noise. This discrepancy may partially be explained by the differences in sample characteristics, statistical power, and analytic approaches, as both prior studies relied on substantially smaller and non-representative samples. The findings from these studies were directionally consistent with our results, although the associations did not reach statistical significance in their categorical (ref. 1) or correlational analyses (ref. 6).

Beyond establishing empirically derived thresholds, several important questions remain regarding the clinical relevance and underlying mechanisms of food noise. First, it is important to characterize the overlap between food noise and related constructs (ref. 7-10), and determine its conceptual boundaries. Second, it is unclear whether food noise is associated with adverse physical, mental, or dysregulated eating outcomes. Establishing such associations will help clarify the extent to which food noise may have clinical implications. Third, the mechanisms that contribute to the development and maintenance of food noise remain unknown. Etiologic research will therefore be important to identify potential treatment targets and informing the development of effective interventions. Finally, given the increasing use of GLP-1 medications and their potential effects on food-related thoughts and appetite, prospective studies should examine the relationship between GLP-1 treatment and food noise.

## Limitations:

This study has several limitations. First, the unidimensional structure of the FNQ limits the extent to which the measure can distinguish among potentially distinct dimensions of food noise. Second, in the CFA, RMSEA exceeded conventional thresholds for acceptable fit and should be interpreted alongside other fit indices given its sensitivity to models with few degrees of freedom (ref. 33). Third, food noise endorsement can be operationalized in multiple ways, and no empirically established threshold currently exists. Nevertheless, our findings provide preliminary evidence to inform the development of clinically meaningful thresholds for defining foods noise. Finally, the cross-sectional design limits causal inference regarding food noise and weight status.

## CONCLUSION

Widespread use of GLP-1 medications has revealed not only their effectiveness in reducing appetite and food intake, but also the clinical relevance of a seemingly underrecognized cognitive-affective process, food noise, that may be highly distressing for some individuals (ref. 5). Although the concept has gained increasing attention in public discourse, empirical research on food noise remains limited. Our study extends this literature by providing an initial estimate of the population scope of food noise endorsement among U.S. adults, including those with and without obesity, and by identifying its associations with sociodemographic characteristics and weight status. These findings have theoretical and clinical implications, while highlighting the need for further research to establish the clinical relevance, underlying mechanisms, and distinctiveness of food noise from related constructs.

## Supplementary Material

This is a list of supplementary files associated with this preprint. Click to download.

- SupplementalMaterialFoodNoise.docx
- Table1.docx
- Table2.docx
- Table3.docx
- Table4.docx

## Figures and Tables

**Figure 1:**
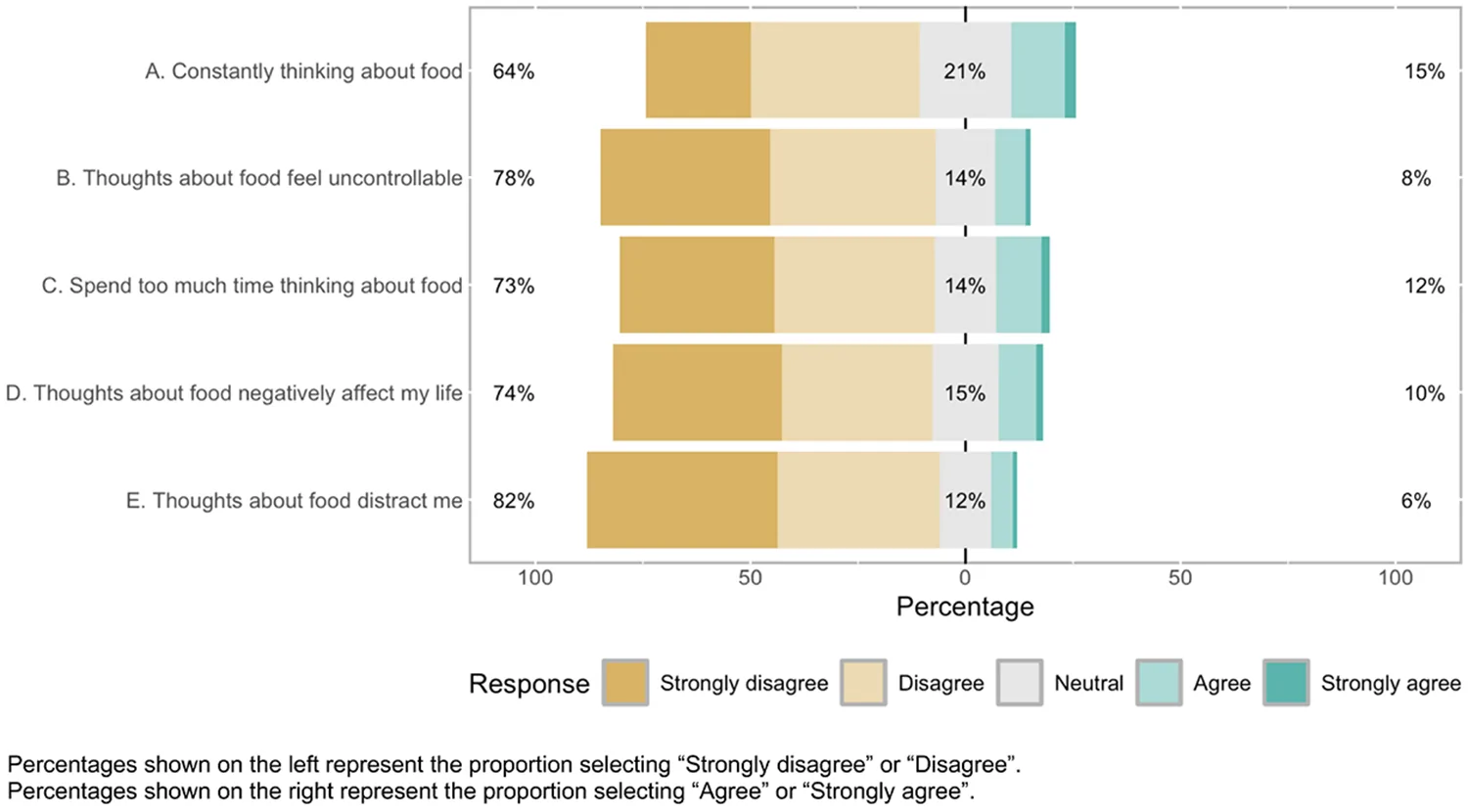
Distribution of responses to Food Noise Questionnaire items

**Table 1.** Polychoric correlation matrix

**Table 2.** Factor Loadings and Confirmatory Factor Analysis Fit Indices for the FNQ

**Table 3.** Prevalence of food noise endorsement by the operational criterion and population

**Table 4.** Demographic characteristics associated with mean Food Noise Questionnaire score

## Data Availability

The datasets generated during and/or analyzed during the current study are not publicly available due to privacy and confidentiality restrictions but are available from the corresponding author on reasonable request.
